# Responsiveness Index versus the RASS-Based Method for Adjusting Sedation in Critically Ill Patients

**DOI:** 10.1155/2021/6621555

**Published:** 2021-10-07

**Authors:** Johanna E. Wennervirta, Mika O. K. Särkelä, Markus M. Kaila, Ville Pettilä

**Affiliations:** ^1^Department of Anesthesiology, Intensive Care and Pain Medicine, University of Helsinki and Helsinki University Hospital, P.O. Box 340, 00029 Helsinki, Finland; ^2^GE Healthcare Finland Oy, Kuortaneenkatu 2, 00510 Helsinki, Finland

## Abstract

**Background:**

Sedation of intensive care patients is needed for patient safety, but deep sedation is associated with adverse outcomes. Frontal electromyogram-based Responsiveness Index (RI) aims to quantify the level of sedation and is scaled 0–100 (low index indicates deep sedation). We compared RI-based sedation to Richmond Agitation-Sedation Scale- (RASS-) based sedation. Our hypothesis was that RI-controlled sedation would be associated with increased total time alive without mechanical ventilation at 30 days without an increased number of adverse events.

**Methods:**

32 critically ill adult patients with mechanical ventilation and administration of sedation were randomized to either RI- or RASS-guided sedation. Patients received propofol and oxycodone, if possible. The following standardized sedation protocol was utilized in both groups to achieve the predetermined target sedation level: either RI 40–80 (RI group) or RASS −3 to 0 (RASS group). RI measurement was blinded in the RASS group, and the RI group was blinded to RASS assessments. State Entropy (SE) values were registered in both groups.

**Results:**

RI and RASS groups did not differ in total time alive in 30 days without mechanical ventilation (*p*=0.72). The incidence of at least one sedation-related adverse event did not differ between the groups. Hypertension was more common in the RI group (*p*=0.01). RI group patients were in the target RI level 22% of the time and RASS group patients had 57% of scores within the target RASS level. The RI group spent significantly more time in their target sedation level than the RASS group spent in the corresponding RI level (*p*=0.03). No difference was observed between the groups (*p*=0.13) in the corresponding analysis for RASS. Propofol and oxycodone were administered at higher RI and SE values and lower RASS values in the RI group than in the RASS group.

**Conclusion:**

Further studies with a larger sample size are warranted to scrutinize the optimal RI level during different phases of critical illness.

## 1. Introduction

Most critically ill mechanically ventilated patients need sedation [1]. Optimized sedation is needed for patient safety and comfort. Sedation practices vary considerably but commonly involve the use of benzodiazepines or propofol and opiates for pain management. Deep sedation is related to prolonged mechanical ventilation, prolonged intensive care unit (ICU) and hospital stay, increased hospital-acquired infections, increased costs, and higher mortality [2–5]. Lighter sedation could improve these outcome measures [6], but it may increase the patients' agitation, discomfort, and long-term psychological disorders [7, 8].

The systematic evaluation of pain, agitation, and delirium in ICU patients is recommended [7], and deep sedation should be avoided. Studies have shown that changing staff attitudes toward lighter sedation may be challenging [9, 10]. Today, sedation is still monitored with clinical assessments like the Richmond Agitation-Sedation Scale (RASS) scoring. RASS differentiates well between arousal to verbal and physical stimulation and uses the duration of eye contact following verbal stimulation as the principal means of titrating sedation [11, 12]. Although RASS is a generally accepted standard assessment method for sedation level, it is not continuous; for every assessment, the patient needs to be stimulated. This stimulation may cause sleep disruption and unnecessary stress. Continuous and objective methods for monitoring sedation are urgently needed in clinical practice.

Electroencephalogram- (EEG-) based devices, primarily developed for depth of anesthesia monitoring, have been studied also as sedation monitors for ICU patients. The frontal electromyogram (EMG) seems to be the major confounding factor for EEG-based monitors in the ICU [13, 14]. Bispectral Index (BIS) and Entropy have been shown to correlate with clinical sedation state, yet they do not discriminate well between different sedation levels [14, 15].

The Responsiveness Index (RI) is a recently described method for ICU sedation monitoring [16], yet it is not commercially available. RI is based on processed frontal EMG, and it is proposed to reflect the interaction between a patient's conscious state and the intensity and frequency of stimulations during treatment [16]. RI uses a time window of the past 60 minutes and creates an index varying between 0 and 100, where low RI values indicate less EMG responses in the past 60 minutes [16]. RI monitor includes “traffic light” coding, where RI values 0–20 are red and correspond to the least responsive patient state, intermediate values 20–40 are amber, and green values 40–100 correspond to awake patients or those exhibiting frequent arousals [17]. In two previous studies, RI has been assessed as a promising tool for monitoring sedation in the ICU [17, 18], and the combination of RI and staff education has been shown to be useful in improving the quality of sedation and analgesia, however with increased sedation-related adverse events [9]. Whereas earlier studies [9, 18] aimed to use RI for avoiding unnecessary deep sedation (RI < 20), RI-controlled sedation has not previously been evaluated at a sedation state (RI > 40), which we supposed to be clinically adequate based on the existing data.

In this open randomized controlled pilot study of 32 critically ill, mechanically ventilated adult patients, we aimed to evaluate the real-time feasibility and efficacy of RI-based sedation compared to standard RASS-based sedation titration. We hypothesized that RI-controlled sedation would be associated with increased total time alive without mechanical ventilation at 30 days without an increased number of adverse events.

## 2. Materials and Methods

**Figure 1:**
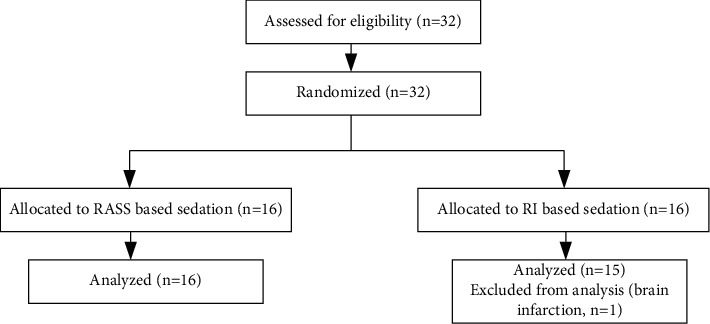
Consort diagram of the study.

The study was conducted in the Helsinki University Hospital, Finland. The Ethics Committee of Helsinki University Hospital approved the study. All the patients, or their next of kin, gave written informed consent to participate and consent for the publication of the data. The study is registered in ClinicalTrials.gov/NCT03250481. This study adheres to CONSORT guidelines.  Figure 1 presents the CONSORT flowchart. Data were gathered between 2013 and 2016.

Patients were randomized into two groups: 16 in the RI group and 16 in the RASS group. Mechanically ventilated patients with estimated continuous sedation exceeding 48 hours (less than 24 hours from ICU admission and less than 12 hours from the start of mechanical ventilation) were included. These time limits were used for securing informed consent and to ensure that the sedation level of each patient is controlled according to the study protocol for the effectual period. Concealed envelopes were used for randomization using a varying block size of 2 to 8. The exclusion criteria were contraindication to propofol or oxycodone as sedatives as assessed by the treating clinician or known or suspected neurological impairment (hypoxic or traumatic brain injury, intracranial hemorrhage, status epilepticus, or drug overdose as an admission diagnosis).

The ICU admits mixed medical-surgical patients, and the nurse to patient ratio for mechanically ventilated patients is 1 : 1.

### 2.1. Sedation Protocol

We randomized the study patients to either RASS target score (−3 to 0) or RI target level of 40–80 for the whole study period. Broad RASS target (−3 to 0) was based on ICU's standardized operative protocol and is a common practice in the clinic. Although we are continually tending to lighter sedation, we realize that some of the patients need deeper sedation and we have accepted RASS −3 to 0 as a suitable target of sedation. RI target was set equivalent to RASS target based on an unpublished statistical analysis of 2722 RI-RASS data pairs collected during the RI development phase and including previous studies [16, 17], where mean (±SD) RI values measured at the time of RASS −3 (*n* = 463), −2 (*n* = 368), −1 (*n* = 383), and 0 (*n* = 232) assessments were 37.3 (±31.6), 41.0 (±30.7), 55.8 (±35.1), and 75.6 (±24.2), correspondingly.

Propofol was infused at an initial rate of 2.4 mg/kg/h for one hour. Thereafter, the infusion rate of propofol was titrated between 0.8 and 4 mg/kg/h to reach or maintain the target RASS score or RI level. If RI or RASS was not at the target or bolus was clinically needed, a propofol bolus of 20–40 mg was administered. If there is persistent severe hypotension, lactatemia, or metabolic acidosis, midazolam could be used instead of propofol. Oxycodone was administered as the first choice as 3–6 mg boluses for pain management. Midazolam was given if the maximum dose of propofol was reached and pain management by oxycodone restricted achievement of the target sedation level. Midazolam was supplied intravenously in boluses of 1–2 mg (based on the weight of the patient), starting at 3 boluses/h for the first hour. Paralytics, dexmedetomidine, or other sedatives were not allowed.

### 2.2. RI and SE Registration

Because we were interested in how the frontal EMG-based RI and electroencephalographic State Entropy (SE) differ as a sedation monitoring tool, both RI and SE data were digitally and continuously registered with E-Entropy monitor (GE Healthcare, Helsinki, Finland) including the RI algorithm in both groups, but in the RASS group, RI and SE values were blinded. Entropy EasyFit Sensor (GE Healthcare, Helsinki, Finland) was applied to the forehead symmetrically relative to the midline. This position was modified from the recommended unilateral position for Entropy monitoring to acquire bilateral frontal EMG data for RI monitoring. Each sensor comprised a strip including one electrode each for the left and right hemisphere and one central ground electrode. Sensors were changed every 24 hours, or in case of a contact issue. The Entropy monitor performed an automatic impedance test every 10 minutes to ensure electrical contact fidelity. According to protocol, data recording of study patients continued until one of the following endpoints was reached:

1. The patient regained consciousness and mechanical ventilation was discontinued
2. 96 hours elapsed from the start-up of registration
3. The patient or a relative requested discontinuation or withdrawal from the study
4. The patient died

### 2.3. RASS Scoring

The study protocol instructed nurses to assess the RASS score hourly in both groups. In the RI group, an assessment was performed by the independent study nurse, who did not participate in the patient treatment, and in the RASS group by the bedside nurse until the end of data recording. The independent study nurse did not register her scoring in the patient data management system or inform the treating clinician or bedside nurse. RASS scores were recorded manually.

### 2.4. Other Data

In addition to RASS, RI, and SE, laboratory data, Sequential Organ Failure Assessment (SOFA) score, sedative drugs, adverse events (AEs), hemodynamic and respiratory physiological parameters, length of mechanical ventilation, ICU length of stay (ICU-LOS), and time of exitus were collected.

### 2.5. Outcomes

The primary endpoint of the study was the incidence of predefined clinical adverse events (AEs) related to sedation or sedation monitoring. Predefined AEs were hypotension (systolic arterial pressure under 90 mmHg), hypertension (systolic arterial pressure over 160 mmHg), tachycardia (heart rate over 100/minute), bradycardia (heart rate under 50/min), tachypnea (breathing frequency over 30/minute), restlessness, unintended catheter removal, gas exchange deficiency, skin irritation caused by electrodes, or hemodynamic instability. The secondary endpoint was total time alive in 30 days without mechanical ventilation. The results of ventilator liberation cover the whole length of stay in ICU, not only the period of study.

### 2.6. Statistical Analysis

As this was a pilot study without adequate knowledge of the incidence of AEs, formal sample size estimation was not done. The secondary endpoint was chosen to avoid bias due to a small sample size, since continuous data generally needs a smaller sample size than binary data, such as mortality. Furthermore, many ICU patients expire related to illness, making mortality a less suitable variable for studying possible differences between sedation monitoring methods.

Compliance to sedation protocol was studied by calculating the percentage of time each patient spent in the RI level below the target (0–39), within the target (40–80), above the target (81–100), or when the monitor did not display a valid value because sensor disconnection or other technical reasons were calculated. Correspondingly, the percentage of given RASS scores below the target (−5 to −4), within the target (−3 to 0), and above the target (1–4) were calculated for each patient. Spearman's rank correlation coefficients between RI vs. RASS and SE vs. RASS value pairs were derived separately for both groups. For avoiding arousal effects caused by the RASS assessment, we used RI and SE values 10 minutes before the time stamp of the RASS assessment. Compliance with sedation protocol was further evaluated by analyzing the RI, SE, and RASS values at the time of administered boluses of propofol and oxycodone. The RI drug effect for propofol and oxycodone was evaluated by comparing RI values at the time of the bolus to RI values 30 minutes before and after the bolus.

Categorical data were compared with Fisher's exact test. Because of the relatively small sample size and for consistency, all quantitative data, except ICU-LOS, were compared by Wilcoxon rank-sum test. The log-rank test was used to compare the ICU-LOS. The level of statistical significance was set to *p* < 0.05. MATLAB 2014b (MathWorks Inc, Natick, MA, USA) was used for statistical analysis, except the log-rank test, which was conducted with open-source R software.

## 3. Results

### 3.1. Patients

After randomization, one RI group patient (of 16) was excluded from the final analysis because of massive brain infarction deemed unrelated to this study. Baseline characteristics are shown in Table 1. SOFA score (at admission) was higher in the RASS than in the RI group (*p*=0.04). Primary diagnoses at ICU admission were pancreatitis in 31% (*n* = 10), pneumonia in 28% (*n* = 9), and other infection in 19% (*n* = 6). 22% (*n* = 7) of the subjects were treated in the ICU postoperatively. A detailed list of ICU admission diagnosis is given in Table 1.

### 3.2. Time Alive in 30 Days without Mechanical Ventilation

The RI and RASS groups did not differ in total time alive in 30 days without mechanical ventilation (Table 2; *p*=0.72). ICU-LOS was comparable between the study groups (Table 2; *p*=0.69). Due to prolonged mechanical ventilation, 6 patients needed tracheotomy (RASS 3/16, RI 3/15).

### 3.3. Adverse Events

The incidence of at least one of the predefined adverse events did not differ between the study groups (Table 2). Hypertension was more common in the RI group (*p*=0.01). Of 31, 8 patients required reintubation (RASS 4/16, RI 4/15). Two unplanned central venous catheter removals occurred, neither of the patients were without an intravenous line after that, and no massive bleeding resulted in catheter removal.

### 3.4. Compliance to the Sedation Protocol

In median, RI group patients spent 22% of the time in the target RI level, and RASS group patients had 57% of their scores in the target RASS level.

**Figure 2:**
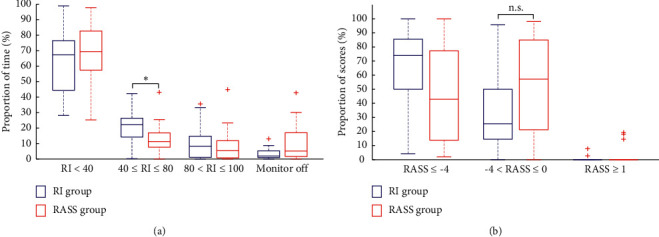
(a) Proportion of monitoring time spent in different RI levels in RI group of patients (blue) and RASS group of patients (red). (b) The proportion of RASS scores. “Monitor off” indicates technical problems, most often sensor contact issues. The horizontal lines within the boxes represent 25%, 50%, and 75% percentiles, the whiskers extend the most extreme data point within 1.5 times the interquartile range, and the crosses represent statistical outliers. ^*∗*^*p* < 0.05; n.s. = no statistical significance.

RI group patients spent significantly more time in their target RI level (40–80) than RASS group patients spent in the corresponding RI level (medians 22% vs. 11%, *p*=0.03) (Figure 2(a)). No difference was observed between the study groups when comparing groups between the times spent in RASS levels −3 to 0 (medians 25% vs. 57%, *p*=0.13) (Figure 2(b)).

**Figure 3:**
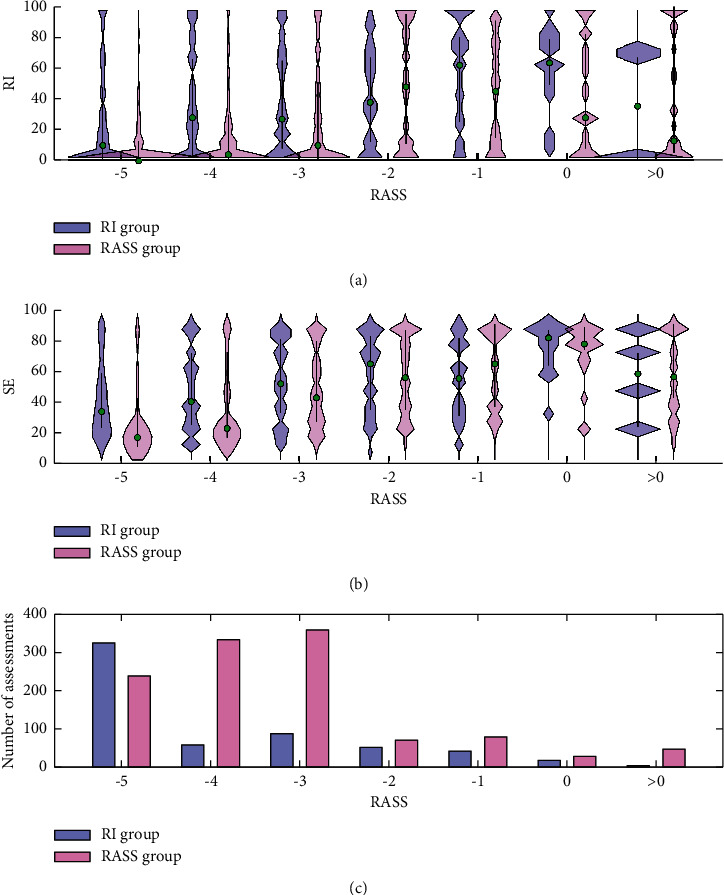
(a) Violin plot presenting RI value distributions at the time of each RASS observation in the RI group (cyan) and in the RASS group (magenta). (b) Violin plots presenting SE value distributions at the time of each RASS observation in the RI group (cyan) and in the RASS group (magenta). (c) Histogram presenting distribution of RASS values in the RI group (cyan) and in the RASS group (magenta). The violin plots present rotated data distribution; i.e., the wider the area is, the more samples are in the range. The thick black line indicates interquartile range, and the green circle is the median.

Spearman's rank correlation coefficients for RI vs. RASS data pairs were 0.34 in the RI group and 0.45 in the RASS group, and those for SE vs. RASS correlations were 0.31 and 0.52, correspondingly. In the RI group, all 587 RASS assessments were done by the independent study nurse, whereas 1160 RASS assessments of the RASS group were done by a bedside nurse.  Figure 3 presents violin plots of RI-RASS data pairs in both groups.

**Figure 4:**
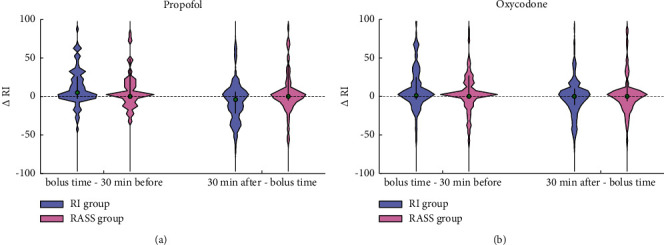
(a) Violin plot presenting the RI value change from 30 minutes before propofol bolus administration to the time of bolus administration (left) and the RI value change from the time of bolus to 30 minutes after the bolus (right). (b) Violin plot presenting the RI value change from 30 minutes before oxycodone bolus administration to the time of its administration (left) and the RI value change from the time of bolus to 30 minutes after the bolus (right). Cyan presents the RI group and magenta the RASS group. The violin plots present rotated data distribution; i.e., the wider the area is, the more samples are in the range. The thick black line indicates interquartile range, and the green circle is the median. In the RI group, propofol boluses were mostly administered to increasing RI values, and the RI values decreased during the following 30 minutes in most cases.

Propofol and oxycodone were administered at higher RI and SE levels and lower RASS values in the RI group than in the RASS group. One patient was treated with midazolam due to severe pancreatic and very high triglyceride values. One patient received a fentanyl infusion instead of oxycodone due to severe circulatory depression and septic shock.  Table 3 presents the RI, SE, and RASS values at the time when a bolus dose was administered. In the RASS group, propofol and oxycodone were administered at lower RI and SE, but higher RASS levels.  Figure 4 illustrates the effect of propofol and oxycodone boluses on RI in both groups. In the RI group, propofol boluses were mostly administered during increasing RI values, and RI values had decreased during the following 30 minutes in most cases. The RI and SE data from one RI group patient were lost and, thus, data of 14 RI group patients were used in this part of the analysis.

## 4. Discussion

In this pilot study, 32 critically ill adult patients were randomized to either RI or RASS-guided sedation. We aimed to evaluate the real-time feasibility of RI-based titration of sedation. We hypothesized that RI-guided sedation would be associated with increased total time alive without mechanical ventilation at 30 days without an increased number of adverse events.

RI-guided sedation did not affect the 30-day total time alive without mechanical ventilation. There was no difference in the number of patients with at least one AE when adding all AEs together between the studied groups. Among the individual adverse events, hypertension was significantly more common in the RI group. Of the 6 hypertensive patients in the RI group, 5 did not have hypertension prior to ICU admission, and they were transferred to follow-up care with antihypertensive medication. An increased risk of hypertension may indicate a more challenging sedation control in the RI group.

Compliance with the sedation protocol was deemed to be poor in both groups. Direct comparison of these percentages is not meaningful: the personnel's routine was to use RASS in their clinical work, whereas RI was introduced to the clinic for the first time during this study. In the RASS group, the targeted sedation level was achieved for 57% and in the RI group for 22% of the time. In both groups, patients tend to be oversedated (Figure 2). A remarkable amount of propofol and oxycodone boluses were given at the deeper sedation level than the target (Table 3). In the RASS group, RASS scores at the time propofol and oxycodone boluses were slightly better in line with the protocol, but the corresponding median SE value of 30 indicated that the sedation was probably unnecessarily deep in that group, as well. As our results indicate, sedation control is not a straightforward task with either of the studied methods.

In this study protocol, we were not able to set a clinically reachable sedation target. In particular, in the RI group, there were difficulties in keeping patients in a state of stable sedation. Thus, it seems plausible that the study setting was suboptimal to compare these two groups. First, the probable effect of education and adaptation should not be underestimated. It would have been useful to introduce RI to the clinic some time before the study and to give personnel the opportunity to familiarize themselves with the new method. Second, our protocol was perhaps too simplified. We followed the traffic light coding of the RI device and set the target for “green” for the whole study period. At least for the early phases of ICU treatment, it may have been better to set the RI target at a lower level, such as avoiding “red,” as was done in the earlier study [18]. Staff education and user adaptation could possibly improve the target precision for RI in the long-term use, and possible experience obtained with only 16 patients was potentially not enough for successful sedation titration.

One problem related to RI use may be that it uses a rather long historical time window for index value calculation, even up to 60 minutes. This may make its response time rather slow. Without knowledge of this feature, the user may become impatient and overshoot titration, leading to a fluctuating level of sedation. This may explain the lower correlation coefficient between RI and RASS pairs in the RI group. On the other hand, nurses commented that sometimes even minor decreases of propofol caused unexpected high increases to RI values. The root cause and prevalence of this phenomenon should be studied in a specific setting, where the time period following the propofol decrease step is expected to be stimulus free. In the RI group, propofol and oxycodone boluses were generally given when the RI displayed a higher value than it had 30 minutes earlier. Encouragingly, the RI demonstrated response 30 minutes after the propofol bolus. Boluses are often administered when increased patient stimulation is expected and that may explain why RI increased for nearly half of the propofol boluses.

Because of the known significant overlap of RI values within RASS levels −4 to 0 [17], it is not possible to define equal target sedation levels for RI and RASS groups. Additionally, we calculated ROC statistics in the RASS group using RASS < −3 as a positive condition. Using the cut-off value of RI < 40, we obtained a sensitivity of 80.3%, a positive predictive value (PPV) of 57.8%, a positive likelihood ratio of 1.40, and an *F*_1_ score of 67.2%. For a cut-off value RI < 20, the measurements were 72.0%, 60.2%, 1.54, and 65.6%, respectively. As these values indicate, even though RI is quite sensitive in the detection of deepest RASS levels, its accuracy is only moderate, because of the large number of false positives. Switching between cut-off values of 40 and 20 did not have an effect to this property. For SE < 40, corresponding ROC statistics were 65.3%, 64.7%, 1.87, and 65.0%; even though the sensitivity of EEG-based sedation measure might be slightly less, the accuracy was on the same level.

State Entropy seems to provide a more linear relationship with sedation level than RI (compare Figures 3(a) and 3(b)). Despite notable overlaps, median SE values consistently increase with increasing RASS from −5 to 0. In the RASS group, RI values were mostly around 0 or close to 100. The target RI range for this study was set based on the mean RI values at the RASS levels −3 to 0. However, the mean value may only reflect how the bimodal RI distribution is divided between very low and very high values. This makes the study setting problematic, because in practice the RI target range of 40–80 may be difficult to achieve. Also, nurses stated that RI values tended to be set as either 0 or 100. However, Figures 2(a) and 3(a) illustrate that more middle-range RI values were achieved when RI was used as a sedation target, especially when RASS was −4 to −3. In the RASS group, the RASS score was observed by the currently attending bedside nurse, whereas in the RI group the RASS score was carried out by the study nurse. All the study nurses were instructed to RASS scoring and they do use it in daily practice. However, as we do not know the interrater reliability of RASS assessments, the control arm of the study should be considered as a representation of the standard practice of the clinic. Also, interobserver variability may explain differences between the left and the right columns of Figure 3.

One recent study [19] with awakening anesthesia patients revealed the possible shortcomings of EMG-based sedation measurements. During the emergence phase of anesthesia, the onset of EMG activity appeared independently from the return of consciousness, and this discrepancy was larger with the patients having an endotracheal tube than with the laryngeal mask patients. In particular, intubated patients had EMG activity despite high effect-site volatile gas anesthetic concentrations. Furthermore, at the onset of EMG activity, 79% of the patients had slow-wave delta EEG activity. The authors concluded that the appearance of EMG after anesthesia activity is commonly occult, and it is not usually linked with cortical arousal. This observation challenges the use of EMG-based RI for propofol titration.

High false-positive rates of RI have also been observed by Walsh et al. [17]. They found it to be acceptable if RI would only be used as a prompt to clinical staff to make a clinical assessment of sedation requirements with a goal to decrease sedation. Results of our study indicate that a low RI value alone is not an adequate criterion for drug dosing, as low RI values (despite moderate or light sedation, RASS > −4) possibly contributed to unnecessary drug adjustments, thus making sedation more unstable. Another recent study [20] demonstrated that the number of false positives could be decreased by combining a baseline EEG with responsiveness information. Wang et al. [20] used the combination of baseline BIS (minimum BIS within 15 minutes before the RASS) and stimulated BIS (maximum BIS within 15 minutes after the RASS). This addition increased the specificity and the PPV when compared to baseline BIS or stimulated BIS alone.

Despite this study and earlier works of Walsh and team [9, 18], there are very few prospective, randomized controlled trials where EEG/EMG derived index is used as an adjunct to sedative drug administration. Weatherburn et al. [21] studied 50 mechanically ventilated surgical and general ICU patients allocated to BIS monitoring and standard monitoring groups. Targeting to BIS > 70 did not show up the difference in morphine or midazolam use, the length of mechanical ventilation, or the ICU stay. Olsen et al. have completed two RCTs [22, 23] with BIS augmented sedation protocol. The first study [22] with 67 neurological patients compared the standard group targeted to Ramsay scale 4 to BIS augmented group, which was first targeted Ramsay 4 and further fine-tuned to a BIS range of 60–70. During the test period of one nursing shift (12 hours), the BIS augmented group received less propofol and woke up quicker after the sedation was turned off for the neurological examination. The latter study [23] with 300 ICU patients compared RASS −2 to BIS augmentated (60–70) RASS −2. The results were contradictory, as BIS augmentation reduced propofol and fentanyl consumption but increased dexmedetomidine and benzodiazepine usage.

Relatively few RCT studies indicate that the challenges related to incorporating EEG indices, which were developed as anesthesia tools, to ICU clinical practices are well recognized. More studies are needed to define a target range of index values in critically ill patients, remembering that different sedatives have different EEG characteristics even though the sedative effect (decreased responsiveness) would be the same. Contrary to BIS and Entropy which was developed using anesthetized patient data, RI has been developed using the data of ICU patients [16]. However, clinical evidence of the usefulness of RI measurement is still limited, and our observation of the possible association between RI-controlled sedation and hypertension should be carefully evaluated in future studies.

The major limitations of our pilot study were the small sample size and slow patient enrollment. The data was collected for almost three years; the main obstacle was the renovation of the surgical part of the ICU. Even though study nurses and treatment protocol did not change during the patient enrollment, the infrequent use of RI monitoring probably did not favor the adoption of new technology.

The interpretation of the results of this pilot study was limited because of the small sample size. Time alive in 30 days without mechanical ventilation did not differ between the studied groups. Thus, even though we had difficulties in titrating sedation in the RI group, we conclude that further studies are warranted to examine the possible clinical benefit of RI. RI and RASS are conceptually two different measurements. The advantage of RI over RASS is as follows: it is providing continuous and objective data that may be useful in clinical decision-making. Thus, RI-guided sedation specifically targeted to the treatment periods and severity of illness should be evaluated in further larger clinical studies.

## 5. Conclusions

We did not find any difference in the proportion of sedation-related adverse events between the RI- or RASS-guided sedation. However, hypertension was more common with RI-guided sedation. Further, larger studies targeting different RI levels considering the phase and severity of critical illness are warranted.

## Figures and Tables

**Table 1:** Baseline characteristics.

	RI group	RASS group	*p* value
No. of males, *N*	12/15	11/16	0.69
ICU admission diagnosis			
Pneumonia	5/15	4/16	0.70
Pancreatitis	4/15	6/16	0.70
Abscess, cellulitis	2/15	1/16	0.60
Sepsis	1/15	0/16	0.48
Peritonitis	0/15	1/16	1.00
Thyroid or tongue tumor	1/15	3/16	0.60
Bowel ischemia	1/15	0/16	0.48
Lung cancer	0/15	1/16	1.00
Lung thrombosis	1/15	0/16	0.48
Age (years)	54 (26–79)	55.5 (20–73)	0.84
Weight (kg)	86 (64–140)	82.5 (57–126)	0.69
Admission SOFA	4 (0–9)	6 (1–12)	0.04^*∗*^
Lactate (mmol/l)	1.3 (0.7–2)	1.35 (0.7–14.8)	0.68
Leukocytes (10^9^/l)	10.9 (5–38.2)	11.95 (1.5–30.9)	0.35
CRP (mg/l)	306 (5–478)	162.5 (25–416)	0.24

Data are presented as the number of patients or median (range). Admission SOFA: Sequential Organ Failure Assessment on admission, CRP: C-reactive protein. ^*∗*^*p* < 0.05; *p* value was estimated with Fisher's exact test for categorical data and Wilcoxon rank-sum test for ordinal data.

**Table 2:** Outcome and adverse event data.

	RI group	RASS group	*p* value
Median days alive in 30 days without mechanical ventilation (range)	7.90 (0–28.82)	15.23 (0–29.25)	0.72
Median ICU length of stay in hours (range)	416 (101–938)	302 (73–1204)	0.69
At least one of the predefined adverse events	12/15	11/16	0.69
Hypertension	6/15	0/16	0.01
Hypotension	1/15	4/16	0.33
Tachycardia	4/15	6/16	0.70
Tachypnea	1/15	2/16	1.00
Unplanned removal of catheter	2/15	0/16	0.23
Restlessness	8/15	5/16	0.29
Other adverse events	3/15	5/16	0.69

Adverse event data are presented as the number of patients with at least one adverse event related to sedation. Other adverse events are gas exchange deficiency, minor skin irritation caused by electrodes, hemodynamic instability, and unplanned extubation. Wilcoxon rank-sum test was used for days alive, log-rank test for ICU length of stay, and for the other data, *p* values were estimated with Fisher's exact test.

**Table 3:** RI, SE, and RASS at the time of propofol and oxycodone boluses.

	RI group	RASS group	*p* value
*Propofol*			
No. of boluses, *N*	261	201	
RI	32.5 (0–100)	12.5 (0–100)	<0.001
SE	55 (5–92)	30 (3–91)	<0.001
RASS	−4 (−5 to 1)	−3 (−5 to 2)	<0.001
*Oxycodone*			
No. of boluses, *N*	313	328	
RI	33 (0–100)	10 (0–100)	<0.001
SE	51 (8–91)	30 (2–92)	<0.001
RASS	−4 (−5 to 1)	−3 (−5 to 2)	<0.001

Presented values are medians (range) of RI, SE, and RASS samples collected at the time when bolus doses of propofol or oxycodone were administered intravenously. RI: Responsiveness Index, SE: State Entropy, RASS: Richmond Agitation-Sedation Scale. One patient in the RASS group received midazolam instead of propofol, and one patient in the RASS group received fentanyl infusion. These patients were excluded from this analysis.

## Data Availability

Data availability is reliant on patient consent forms and juridical clauses, and data can be requested from the corresponding author.
